# A 2-year point-prevalence surveillance of healthcare-associated infections and antimicrobial use in Ferrara University Hospital, Italy

**DOI:** 10.1186/s12879-020-4791-8

**Published:** 2020-01-23

**Authors:** Paola Antonioli, Niccolò Bolognesi, Giorgia Valpiani, Chiara Morotti, Daniele Bernardini, Francesca Bravi, Eugenio Di Ruscio, Armando Stefanati, Giovanni Gabutti

**Affiliations:** 1 Department of Hospital Hygiene & Healthcare-Associated Infection Risk Management, Hospital Health Medical Management, S. Anna University Hospital of Ferrara, Ferrara, Italy; 2 Postgraduate School of Hygiene and Preventive Medicine, University of Ferrara, Ferrara, Italy; 3 Research Innovation Quality and Accreditation Unit, S. Anna University Hospital of Ferrara, Ferrara, Italy; 4 Medical Director, S. Anna University Hospital of Ferrara, Ferrara, Italy; 5 Department of Medical Sciences, University of Ferrara, via Fossato di Mortara 64b, 44121 Ferrara, Italy

**Keywords:** Infection control, Cross infection, Iatrogenic disease, Antimicrobial stewardship, Drug resistance, bacterial, Point prevalence survey

## Abstract

**Background:**

Healthcare-Associated Infections (HAIs) represent one of the leading issues to patient safety as well as a significant economic burden. Similarly, Antimicrobial Use (AMU) and Resistance (AMR) represent a growing threat to global public health and the sustainability of healthcare services.

**Methods:**

A Point Prevalence Survey (PPS) following the 2016 ECDC protocol for HAI prevalence and AMU was conducted at Ferrara University Hospital (FUH). Data were collected by a team of trained independent surveyors in 2016 and 2018. Risk factors independently associated with HAI were assessed by a multivariate logistic regression model.

**Results:**

Of the 1102 patients surveyed, 115 (10.4%) had an active HAI and 487 (44.2%) were on at least 1 systemic antimicrobial agent. Factors independently associated with increased HAI risk were a “Rapidly Fatal” McCabe score (expected fatal outcome within 1 year), presence of medical devices (PVC, CVC, indwelling urinary catheter or mechanically assisted ventilation) and a length of hospital stay of at least 1 week. The most frequent types of HAI were pneumonia, bloodstream infections, and urinary tract infections. Antimicrobial resistance to third-generation cephalosporins was observed in about 60% of *Enterobacteriaceae*.

**Conclusions:**

The survey reports a high prevalence of HAI and AMU in FUH. Repeated PPSs are useful to control HAIs and AMU in large acute-care hospitals, highlighting the main problematic factors and allowing planning for improvement actions.

## Background

Healthcare-associated infections (HAIs) represent one of the leading issues to patient safety as well as a significant economic burden on healthcare systems [1].

About 3.2 million HAIs occur every year in the European Union (EU) [2], causing 37,000 deaths as a direct consequence [3], over 2.5 million Disability Adjusted Life Years (DALYs) [4] and 16 million extra days of hospitalization, with an approximate cost of around 7 billion euros [3]. In 2016, the European Centre for Disease Prevention and Control (ECDC) estimated that the burden of six most frequent types of HAI (pneumonia, urinary tract infection – UTI, surgical site infection, *Clostridium difficile* infection) in the EU was higher than the combined impact of 31 other infectious diseases under ECDC surveillance [5].

In the USA, it has been estimated that the 5 most impacting HAIs have a cost of 9.8 billion dollars: surgical site infections, Ventilation-Associated Pneumonia (VAP), Central Venous Catheter (CVC) associated bloodstream infections, *C. difficile* infections, and UTIs accounting for 33.7, 31.6, 18.9, 15.4 and < 1% of total costs, respectively [6].

In Italy, about 450–700 thousand HAIs occur every year in hospitalized patients, of which 30% considered avoidable. Currently, an HAI monitoring system is still not available at national level, and related data are limited to prevalence studies [7].

Similarly, antimicrobial use (AMU) and resistance (AMR) represent a growing threat to global public health and the sustainability of healthcare services [8]. The spreading of carbapenemase-producing Enterobacteriaceae since 2010 is associated with high lethality in hospital settings [9] and a global alert has been raised by the World Health Organization (WHO) [10]. A systematic review of infections with antibiotic-resistant bacteria in the EU estimated about 670,000 cases in 2015, of which 63.5% were associated with healthcare. About 33,000 deaths and 870,000 DALYs were attributable to these infections [11].

Italy is one of the European countries with the highest level of AMU and with the highest prevalence of AMR both in the community and in hospital settings [12, 13]. In 2015, the project “Good practices for the surveillance and control of antimicrobial resistance” was funded by the Italian National Centre for Disease Prevention and Control (CCM) in order to promote integrated actions at national level to control AMR [14].

In order to estimate the burden of HAIs and AMR in the EU, ECDC provided a standardized protocol for HAI Point Prevalence Survey (PPS) to allow data gathering and comparison. The first ECDC prevalence study in the EU was realized in 2011–2012 [2]. A second ECDC study (PPS2) has been conducted in 2016–2017 [13]. In Italy, HAI prevalence in PPS2 (2016) resulted equal to 8.0% [13, 15]. The most common infections were pneumonia (20.3%), bloodstream infections (18.3), UTI (18%), surgical site infections (14.4%) and gastrointestinal infections (8.5%).

In Ferrara University Hospital (FUH), HAI and AMR monitoring have begun in 1992. In 2011 FUH has joined ECDC PPS. An internal survey was repeated with the same protocol in 2012 and 2013 and data were published [16]. Subsequently, FUH also participated in 2016–2017 ECDC PPS. Additionally, FUH implemented for a long time risk management and infection control actions, including surveillance of surgical site infections, UTI and bloodstream infections, antimicrobial stewardship [17] and an AMR alert system, retraining of healthcare workers and application of WHO Guidelines on hands hygiene [18]. In 2018 a Lean Healthcare Management program for infection control (Lean4Health) was adopted in FUH, establishing improvement actions in Rehabilitation and Surgical departments [19].

The objective of this study was to determine the (1) prevalence of HAI and associated risk factors, (2) distribution of systemic AMU and AMR trend in FUH, comparing prevalence data recorded in 2016 and 2018.

## Methods

The point prevalence survey took place from November 14th through 30th of 2016 and from November 12th through 23rd of 2018 in FUH, a tertiary care hospital in Northern Italy accounting 658 beds (578 acute-care, 70 rehabilitation, 10 post-acute care).

Study protocol was approved by the Independent Ethical Committee of Area Vasta Emilia Centrale (CE-AVEC, study code: 638 t2018/Oss/AOUFe; date of approval CE: 17/10/2018). As no information which may identify the subjects was collected, no informed consent was obtained.

Both surveys followed the ECDC PPS protocol version 5.3 [20]. In order to eliminate potential sources of conflict of interest, under-reporting of HAIs and variability of results, data were gathered by trained, independent surveyors (resident doctors of the Postgraduate School of Hygiene and Preventive Medicine of University of Ferrara) and one survey leader (Infection Risk Manager of FUH), consulting clinical documentation and interviewing Medical and Assistant Referents for the management of infectious risk in each department.

All inpatients and newborns admitted to the wards before 8:00 AM and not discharged at the time of the survey were included as indicated by the ECDC protocol [20]. Patients admitted to the emergency department and day surgery were excluded. Data collection in each ward was completed within the same day.

Data included admission date, patient demographics, systemic antimicrobial therapy, active HAIs, surgical procedures (using National Health Safety Network – NHSN categories [20]) and medical devices presence at the time of survey or on the HAI onset date (urinary catheter, CVC, peripheral venous catheter – PVC, mechanical ventilation), antimicrobial resistance data when blood culture was available and patients underlying medical conditions through McCabe score [21]. McCabe score is a classification of the severity of patient’s comorbidities, including chronic conditions and conditions impairing immunological system (for example diabetes requiring amputation or post amputation, end-stage hematological malignancies, chronic leukemias, metastatic carcinoma).

Active HAI definition required infection symptoms on the survey day or systemic antimicrobial treatment on the survey day for symptoms present previously and to meet ECDC surveillance criteria for HAI [20]. Antimicrobials have been categorized accordingly to the Anatomical Therapeutic Chemical classification (ATC) [22].

Time at risk is the time in days elapsed from the date of admission to the date of the survey. It has been categorized in 0 (0–6 days) and 1 (> 6 days). The cutoff of 6 days has been chosen accordingly to the median time of hospital stay on survey date resulted in the Italian PPS2 Report 2016/2017 [15].

### Statistical analysis

Categorical data were expressed as total numbers and percentages. The Shapiro-Wilk test was used to test for normality of the distribution of the continuous variables. In the presence of symmetry of the distributions, the variables will be represented with the mean and Standard Deviation (SD) or, in the case of non-normal distribution, with the median value and Interquartile Range [IQR – 1st quartile 3rd quartile]. Statistical comparisons of categorical variables were assessed using Pearson’s χ2 test or Fisher’s exact test depending on the minimal expected count in each crosstab. Unadjusted logistic regression analysis was performed to estimate Odds Ratios (ORs) and respective 95% Confidence Intervals (95% CIs). All variables were allowed entry in the multivariate logistic regression model, including those that resulted statistically not significant in the univariate analysis. The final multivariate logistic regression model has been estimated using a backward elimination stepwise procedure with a significance level of *p* < 0.05 for a variable to stay in the model. Model calibration was assessed using the Hosmer-Lemeshow goodness-of-fit test [23]. Area under the Receiver-Operating Curve (AROC) was used to assess discrimination power of the model.

Data was recorded with ECDC provided software HELICSWin.net v.1.3. All analyses were performed using STATA/SE ver 13.1 (Stata Corporation, College Station, Texas, USA). *P*-value < 0.05 was defined as statistically significant.

## Results

### Patient baseline characteristics

A total of 1103 patients were included in the two surveys; 1 patient was excluded for incomplete data entry. Therefore, 1102 patients were included in the final analysis (530 in 2016, 572 in 2018). The demographics and clinical characteristics of patients are summarized in Table 1. The median age was 71 [IQR 56 82] years and the median duration of hospital stay was 7 [IQR 3 15] days.

**Table 1:** Baseline Characteristics and Risk Factors for Healthcare-Associated Infections

			Unadjusted logistic regression			Adjusted logistic regression⁑			
Patient Characteristics	Patients without HAI	Patientswith HAI		95%	CI		95%	CI	
	*n* = 987	*n* = 115	OR	lower	upper	OR	lower	upper	*p*-value
Sex									
female*, n (%)*	498 (50.5)	57 (49.5)	1.04	0.70	1.52				
Age, years									
median [IQR]	71 [56 82]	71 [57 82]	1.01	0.99	1.01				
Age classes*, n (%)*									
15–64 years	345 (35.0)	39 (33.9)	Ref						
0–1 years	29 (2.9)	3 (2.6)	0.92	0.27	3.14				
2–14 years	15 (1.5)	0 (0.0)	–	–	–				
> 64 years	598 (60.6)	73 (63.5)	1.08	0.72	1.63				
McCabe Score*, n (%)*									
Non-fatal disease	594 (61.0)	30 (27.0)	Ref						
Ultimately fatal disease	233 (24.0)	35 (31.5)	**2.97**	1.78	4.96				
Rapidly fatal disease	146 (15.0)	46 (41.5)	**6.23**	3.80	10.22	**2.63**	1.59	4.36	0.001
Medical Devices*, n (%)*									
Presence of PVC	674 (68.3)	81 (71.7)	1.18	0.76	1.81	**2.80**	1.57	4.99	0.001
Presence of urinary catheter	346 (35.2)	73 (64.6)	**3.36**	2.24	5.05	**1.78**	1.10	2.90	0.019
Presence of CVC	120 (12.2)	52 (45.2)	**5.96**	3.94	9.02	**3.59**	1.98	6.51	< 0.001
Presence of MV	18 (1.8)	17 (14.9)	**9.43**	4.71	18.90	**4.13**	1.68	10.19	0.002
Surgery*, n (%)*									
None	688 (69.9)	75 (65.2)	Ref						
Non-NHSN	148 (15.0)	19 (16.5)	1.18	0.69	2.01				
NHSN	149 (15.1)	21 (18.3)	1.29	0.77	2.16				
Time at risk*, days									
median [IQR]	6 [2 13]	15 [9 29]	**1.01**	1.003	1.011				
Time at risk*, n (%)*									
> 6 days	472 (47.8)	103 (89.6)	**9.37**	5.08	17.24	**9.38**	4.87	18.08	< 0.001
Year of the survey*, n (%)*									
2016	478 (48.4)	52 (45.2)	Ref						
2018	509 (51.6)	63 (54.8)	1.14	0.77	1.68				

Abbreviations: *HAI* Healthcare-Associated Infection, *IQR* Interquartile Range, *PVC* Peripheral Vascular Catheter, *CVC* Central Venous Catheter, *MV* Mechanical Ventilation, *NHSN* National Health Safety Network, *OR* Odds Ratio, *CI* Confidence Interval, *Ref* Reference Category

*Time elapsed from date of admission to date of survey

⁑Model log-likelihood = − 265.84; LR χ2 = 170.8, *p* < 0.001. Hosmer-Lemeshow χ2 = 6.81, *p* = 0.4486. Area under receiver operating characteristics curve (AROC) = 0.84. Values in bold are statistically significant (*p* < 0.05)

Of the patients surveyed, 337 (30.5%) underwent at least one surgical procedure since admission, of which 170 (50.4%) had major surgeries according to ECDC criteria.

### Factors associated with HAIs

In the unadjusted analysis patients with worst McCabe Scores (Ultimately Fatal and Rapidly Fatal disease), that had CVC, indwelling urinary catheter, mechanically assisted ventilation or hospitalized for more than 6 days since admission were at an increased risk of HAI. (Table 1).

Multivariate logistic regression showed that patients with “Rapidly Fatal” McCabe Scores, who had PVC, CVC, indwelling urinary catheter or mechanically assisted ventilation, or hospitalized for more than 6 days since admission were at an increased risk of HAI. (Table 1).

### Prevalence of HAIs

Overall HAI prevalence was 10.4% (patients with at least one HAI), 10.0% in 2016 (53 patients) and 11.0% in 2018 (63 patients). In acute-care wards HAI prevalence resulted 9.9% in 2016 (9.2% in medical departments, 7.6% surgical departments, 35.0% intensive care units) and 11.8% in 2018 (12.4% in medical departments, 6.8% in surgical departments, 36.8% in intensive care units). In rehabilitation wards prevalence resulted 12.1% in 2016 and 5.8% in 2018. No HAIs were present in post-acute care.

Total count of HAIs resulted 63 in 2016 and 74 in 2018. Most common infection types in 2016 were: pneumonia (27.0%), UTI (25.4%), bloodstream infections (15.9%), gastrointestinal infections (9.5%), surgical site infections (6.3%) and clinical sepsis (6.3%). In 2018 were pneumonia (32.4%), bloodstream infections (21.6%), gastrointestinal infections (10.8%), surgical site infections (9.5%), UTI (6.8%). 2016–2018 trends in HAIs resulted statistically significant for UTIs only (*p* = 0.003). See Table 2 for complete results.

**Table 2:** Prevalence, clinical setting, infection site of Healthcare-Associated Infections and Antimicrobial Use by year of survey

	2016	2018	Total
Total No. of Patients, *n (%)*	530 (48.1)	572 (51.9)	1102 (100.0)
HAI Prevalence (at least one), *n (%)*†	52 (9.8)	63 (11.0)	115 (10.4)
Antimicrobials use prevalence (at least one), *n (%)*†	239 (45.0)	248 (42.8)	487 (44.2)
HAI by clinical setting, n (%)*†	52	63	115
Surgery	9 (17.3)	6 (9.5)	15 (13.0)
Medicine	27 (51.9)	40 (63.5)	67 (58.3)
Geriatrics	0 (0.0)	1 (1.6)	1 (0.9)
Intensive Care	7 (13.5)	7 (11.1)	14 (12.2)
Gynaecology/Obstetrics	0 (0.0)	3 (4.8)	3 (2.6)
Paediatrics/Neonatology	1 (1.9)	2 (3.2)	3 (2.6)
Rehabilitation	8 (1.5)	4 (6.3)	12 (10.4)
Post-acute care	0 (0.0)	0 (0.0)	0 (0.0)
Total No. of HAI	63	74	137
*HAI by infection site, n (%)*‡			
Pneumonia	17 (27.0)	24 (32.4)	41 (29.9)
Bloodstream Infections	10 (15.9)	16 (21.6)	26 (19.0)
Urinary Tract Infections‡	16 (25.4)	5 (6.8)	21 (15.4)
Gastro-intestinal system infections	6 (9.5)	8 (10.8)	14 (10.2)
Surgical site infections	4 (6.3)	7 (9.5)	11 (8.0)
Clinical sepsis	4 (6.3)	4 (5.4)	8 (5.8)
Infections of ear, nose, throat, larynx and mouth	1 (1.6)	5 (6.8)	6 (4.4)
Cellulitis, wound, deep soft tissue not involving bone, not related to surgery	0 (0.0)	4 (5.4)	4 (2.9)
Lower respiratory tract infections	2 (3.2)	1 (1.4)	3 (2.2)
Others	3 (4.8)	0 (0.0)	3 (2.2)
Total No. of antimicrobials	348	345	693
*ATC Antimicrobial classes, n(%)*†			
J01CR Combinations of penicillins, incl. Beta-lactamase inhibitors	95 (27.3)	82 (23.8)	177 (25.6)
J01DD Third-generation cephalosporins	72 (20.7)	78 (22.6)	150 (21.6)
J01MA Fluorochinolones	28 (8.0)	24 (7.0)	52 (7.5)
J01FA Macrolides	13 (3.7)	20 (5.8)	33 (4.7)
J02 AC Triazole derivatives	15 (4.3)	17 (4.9)	32 (4.6)
J01DH Carbapenems	16 (4.6)	15 (4.3)	31 (4.5)
J01XD Imidazole derivatives	17 (4.9)	14 (4.1)	31 (4.5)
J01XX08 Linezolid	13 (3.7)	16 (4.6)	29 (4.1)
J01XA Glycopeptide antibacterials	12 (3.4)	15 (4.3)	27 (3.8)
J01DB First-generation cephalosporins	11 (3.2)	14 (4.1)	25 (3.6)
J01FF Lincosamides	9 (2.6)	8 (2.3)	17 (2.5)
J01CA Penicillins with extended spectrum	8 (2.3)	7 (2.0)	15 (2.2)
J01GB Aminoglycosides	12 (3.4)	2 (0.6)	14 (2.0)
A07AA Intestinal anti-infectives antibiotics	0 (0.0)	8 (2.3)	8 (1.1)
J01XX09 Daptomycin	3 (0.9)	4 (1.2)	7 (1.0)
Others	24 (6.9)	21 (6.1)	45 (6.5)

Abbreviations: *HAI* Healthcare-Associated Infection, *AMU* Antimicrobial Use, *ATC* Anatomical Therapeutic Chemical Classification. *Percentages have been calculated on HAI prevalence in 2016 and 2018, respectively

† *p*-value = not significant; ‡ *p*-value < 0.05

### Antimicrobial use

Four hundred eighty-four inpatients (44.2%) were receiving at least one antimicrobial drug, 239 (45.0%) in 2016 and 245 (42.8%) in 2018, respectively. Total prescribed antimicrobial count resulted 348 in 2016 and 345 in 2018. ATC most frequent antimicrobial classes were: “combinations of penicillins, including beta-lactamase inhibitors” (27.3% in 2016 and 23.8% in 2018), “third-generation cephalosporins” (20.7% in 2016 and 22.6% in 2018), “fluoroquinolones” (8.0% in 2016, 7.0% in 2018). 2016–2018 trends in AMU resulted not significant. (Table 2).

### HAI pathogens and antimicrobials resistance

At the time of the surveys, microbiological data were available for 59 HAI (20 in 2016 and 39 in 2018). Most frequent pathogens resulted *C. difficile* (16.9%), *K. pneumoniae* (11.9%), *C. albicans* (8.5%), *E. coli* (8.5%) and *S. epidermidis (*8.5%), *S. aureus* (6.8%), *S. maltophilia* (6.8%). Of these, 60.0% of *E. coli* were nonsusceptible to third-generation cephalosporins. Nonsusceptibility to third generation cephalosporins and carbapenems was present in 57.1 and 14.2% of *K. pneumoniae*, respectively. *S. aureus* was Oxacillin resistant in 1 case (25.0%).

## Discussion

The main results of this study were that 10.4% of inpatients had at least 1 HAI and 44.2% inpatients was on at least 1 systemic antimicrobial agent, without any statistically significant difference observed between 2016 and 2018 surveys. AMU shows a constantly decreasing trend in FUH surveys (54.4% in 2011, 50.1% in 2012, 48.4% in 2013, 45.0% in 2016, 42.8% in 2018, 16].

The prevalence of HAIs observed in FUH resulted higher than in other studies conducted in the EU and based on ECDC protocol. EU corrected HAI prevalence after validation for PPS2 resulted 6.5% in 2016. In this study, reported HAI prevalence for Italy over a sample of 14,476 patients was 8.0%, one of the highest values in the EU countries [13]. However, in PPS2 HAI prevalence was higher in larger hospital accounting more than 500 beds (9.32%) and lower in smaller hospitals (5.97%). The proportion of patients undergoing at least 1 systemic antimicrobial agent was similar (44.5%) [15]. A multicentric PPS study in Switzerland observed an HAI prevalence of 5.6% [24]. The first national PPS in Singapore acute-care hospitals reported similar results for HAI prevalence (11.9%) and a higher AMU (51.0%) [25]. As in other studies, intensive care unit was the clinical setting with higher HAI prevalence (35.0% in 2016 and 36.8% in 2018, 2,15,16,24].

Among all inpatients, factors independently associated with increased HAI risk were a presence of medical devices (PVC, CVC, indwelling urinary catheter or mechanically assisted ventilation) a length of hospital stay of at least 1 week and a “Rapidly Fatal” McCabe score (expected fatal outcome within 1 year).

Regarding the type of HAI, pneumonia, bloodstream infections and UTI were the three most frequent considering both surveys. However, UTI significantly decreased in 2018 (from 25.4 to 6.5%), dropping behind gastrointestinal system infections and surgical site infections. This could be explained by the Lean Healthcare Management program that was carried out in FUH in 2018, establishing improvement actions in Rehabilitation and Surgical departments and the update of FUH standard operating procedure for prevention of catheter-associated urinary tract infection according to the latest available evidence-based guidelines [26–29]. In 2019, the Lean Healthcare Management program has been extended to prevention and management of bloodstream infections in all hospital wards, including the Emergency Department.

The most frequent causative HAI pathogen was *C. difficile* (CD). Infections caused by CD represent one of the 5 most financially impacting HAIs [6]. American College of Gastroenterology “Guidelines for diagnosis, treatment and prevention of CD infections” report that antibiotics are the main risk factor for CD infections, particularly cephalosporin, fluoroquinolones and clindamycin [30]. These three drugs classes represent 30% of all systemic antimicrobials prescribed in FUH. Studies confirm the importance of antimicrobial stewardship, pointing out a reduction in CD infections incidence up to 60% [31–33]. Furthermore, AMR to third-generation cephalosporins (the second most frequently prescribed systemic antimicrobial in FUH) was observed in about 60% of *Enterobacteriaceae* and this family of microorganism was the causative pathogen of 25% of all HAI.

### Limitations

These surveys have some limitations. First, our study considers a large acute-care hospital, therefore results are not generalizable for smaller hospitals (< 500 patients). As pointed out in the second Italian PPS, HAI prevalence may indeed vary greatly with the hospital number of beds and the case-mix [15]. Second, data on AMR are limited because only a small proportion of microorganisms were tested as part of diagnosing.

## Conclusions

Results of this study contribute to reinforce the statement that HAI and AMR remain a high burden for healthcare systems, undermining patient safety in hospitals and causing high rates of morbidity, mortality and costs [6].

Twenty years since the report To Err is Human [34], hospitals still need to be made safer, adopting evidence-based protocols for medical devices management and strict application of infection control guidelines, especially for frail patients with short life expectancy and prolonged hospital stays, in order to prevent HAI, reduce AMU and limit AMR.

PPSs represent a fast, easily repeatable, and not expensive method to accomplish HAI and AMU surveillance in health-care structures, pointing out priority areas that need improvement actions and providing feedback to health care professionals. Furthermore, the ECDC PPS protocol has been used worldwide [25, 35], allowing comparisons among different studies.

## Data Availability

The datasets used and analyzed during the current study are available from the corresponding author on reasonable request.

## Abbreviations

AMR: Antimicrobial Resistance
AMU: Antimicrobial Use
AROC: Area under the Receiver-Operating Curve
ATC: Anatomical Therapeutic Chemical classification
CCM: Italian National Centre for Disease Prevention and Control
CI: Confidence Intervals
CVC: Central Venous Catheter
DALY: Disability Adjusted Life Years
ECDC: European Centre for Disease Prevention and Control
EU: European Union
FUH: Ferrara University Hospital
HAI: Healthcare Associated Infections
IQR: Interquartile Range
MV: Mechanical Ventilation
NHSN: National Health Safety Network
OR: Odds Ratio
PPS: Point Prevalence Survey
PVC: Peripheral Venous Catheter
Ref: Reference Category
UTI: Urinary Tract Infections
VAP: Ventilation Associated Prenumonia
WHO: World Health Organization
